# Construction of an In Vitro Air–Liquid Interface Exposure System to Assess the Toxicological Impact of Gas and Particle Phase of Semi-Volatile Organic Compounds

**DOI:** 10.3390/toxics10120730

**Published:** 2022-11-26

**Authors:** Stephanie Binder, Narges Rastak, Erwin Karg, Anja Huber, Evelyn Kuhn, George C. Dragan, Christian Monsé, Dietmar Breuer, Sebastiano Di Bucchianico, Mathilde N. Delaval, Sebastian Oeder, Martin Sklorz, Ralf Zimmermann

**Affiliations:** 1 Joint Mass Spectrometry Center (JMSC) at Comprehensive Molecular Analytics (CMA), Helmholtz Zentrum München, 85764 Neuherberg, Germany; 2 Joint Mass Spectrometry Center (JMSC) at Analytical Chemistry, Institute of Chemistry, University of Rostock, 18051 Rostock, Germany; 3 Berufsgenossenschaft Handel und Warenlogistik (BGHW), 80639 Munich, Germany; 4 Institute for Prevention and Occupational Medicine of the German Social Accident Insurance (IPA), 44789 Bochum, Germany; 5 Institute of Occupational Safety of the German Social Accident Insurance (IFA), 53757 Sankt Augustin, Germany

**Keywords:** semi-volatile organic compounds (SVOCs), gas phase (GP), particle phase (PP), dibutyl phthalate (DBP), air–liquid interface (ALI), particle-induced oxidative DNA damage

## Abstract

Anthropogenic activities and industrialization render continuous human exposure to semi-volatile organic compounds (SVOCs) inevitable. Occupational monitoring and safety implementations consider the inhalation exposure of SVOCs as critically relevant. Due to the inherent properties of SVOCs as gas/particle mixtures, risk assessment strategies should consider particle size-segregated SVOC association and the relevance of released gas phase fractions. We constructed an in vitro air–liquid interface (ALI) exposure system to study the distinct toxic effects of the gas and particle phases of the model SVOC dibutyl phthalate (DBP) in A549 human lung epithelial cells. Cytotoxicity was evaluated and genotoxic effects were measured by the alkaline and enzyme versions of the comet assay. Deposited doses were assessed by model calculations and chemical analysis using liquid chromatography tandem mass spectrometry. The novel ALI exposure system was successfully implemented and revealed the distinct genotoxic effects of the gas and particle phases of DBP. The empirical measurements of cellular deposition and the model calculations of the DBP particle phase were concordant.The model SVOC DBP showed that inferred oxidative DNA damage may be attributed to particle-related effects. While pure gas phase exposure may follow a distinct mechanism of genotoxicity, the contribution of the gas phase to total aerosol was comparably low.

## 1. Introduction

Semi-volatile organic compounds (SVOCs) encompass a wide range of different compound classes originating from natural and anthropogenic sources. The ubiquitous use of SVOCs in manufacturing processes and consumer goods renders the workplace and indoor environments as particularly relevant with respect to human exposure [1,2]. For instance, phthalates constitute one of the most prevalent indoor pollutants and are concomitantly known to cause severe adverse health effects [3,4]. Increasing evidence suggests that airborne phthalates are drivers for the development and promotion of lung diseases [5]. While aerosol measurement studies mainly focus on qualitative data and total particle mass concentrations of indoor SVOC pollution [1], information on particle size distribution, associated SVOC sorption, and the partitioning of SVOCs between gas phase, airborne particles, as well as settled dust, are important parameters for the prediction of SVOC lung deposition, the setting of limit values, and the associated risk assessment [6,7]. The inhalation toxicity of SVOCs, though gaining health-related interest, is rather scarcely studied due to the high complexity and dynamics of gas/particle partitioning and associated challenges in drawing appropriate dose-related conclusions [8]. A general focus in inhalation toxicology research is set on the effects of the particle fraction, though little is known about the contribution of the distinct phases of air pollutants—with a specific emphasis on the knowledge gap in the role of the gas phase. Due to their lower vapor pressure compared to volatile organic compounds (VOCs), SVOCs are prone to adhere to particulate matter or surfaces and aggregate upon gas phase adsorption, and therefore encounter a total increase in airborne abundance with increased particle concentrations [9,10,11]. Gas/particle partitioning mainly influences the indoor fate of SVOCs and represents an essential parameter for the prediction of human lung deposition [12]. In the respiratory tract, mucus/gas partitioning strongly influences the bioaccessibility of SVOCs, leading to the conclusion that volatility and solubility are crucial parameters regarding inhalation exposure. The inherent property of SVOCs to co-exist in the gas and particle phases simultaneously affects the deposition mechanisms in the respiratory tract [13]. According to Wei et al. (2020), solubility specifically affects pure gas phase deposition in the different respiratory regions. On the other hand, the deposition of the mixed gas and particle phase strongly depends on the respective inhaled mass concentrations and SVOC volatility. While gas phase SVOCs with a low solubility, for instance phthalates, will therefore preferably follow the inhaled airflow and deposit in the deeper lung regions, the fraction of the particles that deposit in the lung depends on particle deposition mechanisms [14]. It is a generally acknowledged theorem that particle surface area, dependent regional lung deposition, and tissue translocation impact the biological response. More than 20 years ago, Johnston et al. (2000) laid an important foundation to differentiate between the gas and particle toxicity of a specific substance by studying the effect of the heat emissions of the frequently used polytetrafluoroethylen (PTFE) on rodents [15]. The outcome of this study highlighted the importance of considering the aggregation state, particle size, and translocation mechanisms for the toxicological risk assessment of a given compound. Ultrafine particles (UFPs) generated from PTFE foams led to strong pulmonary toxicity in the rat lung, while neither the fine particle size range nor gas phase induced a comparable toxic response. An ultimate conclusion of this study was the postulate of higher pulmonary inflammatory responses induced by UFPs compared to larger particles most likely due to increased surface effects/local dose and immunoevasion. However, the question remains to what extent the aggregation state of a given SVOC affects its toxicological impact.

To address the question of the gas versus particle toxicity of SVOCs, we developed a newly designed in vitro air–liquid interface (ALI) exposure system. The properties and determined parameters of the newly developed ALI system and the connected upstream aerosol generation unit are prerequisites for the differentiation between gas and particle effects by ensuring the same amount/dose of DBP in the different physical conditions. Commercial ALI exposure systems that provide for efficient aerosol transfer to the ALI cell layer are frequently based on stagnation flow exposure, which leads to a direct contact of the aerosol flow with the cells. The humidified laminar aerosol flow is guided towards the ALI cell layer through a trumpet-shaped inlet. The perpendicular flow results in a uniform aerosol distribution, homogeneous and well-defined dosimetry, and a high exposure efficiency according to the sedimentation and diffusion mechanisms of the gas and particle phases [16,17,18]. While ALI exposure system applications focus on particle-induced toxicity [16], the implementation of ALI systems that address the toxicity and deposition efficiency of gaseous pollutants is gaining increasing attention [18,19,20]. However, it is of utmost importance to consider both gas and particle phase exposure to chemical pollutants and associated adverse health effects. Disregarding one of the phases from toxicological risk assessment may significantly underestimate potential contributions to adverse health effects [21]. For that purpose, we designed a new ALI system that allows for separate exposure to the gas and particle phases of SVOCs and therefore distinguishes the respective inferred toxic effects. The simple and short tubing alignment and the straightforward aerosol humidification in the new system allow for well-controlled exposures and gas/particle phase dosimetry, and a thorough aerosol characterization directly before it extends to the ALI module exposure unit. Additionally, the modular set-up adopted from commercial ALI exposure systems permits the combinations of the ALI system with the aerosol emission sources of distinct origin.

Our previous experiments showed that the extensively used SVOC dibutyl phthalate (DBP) induces genotoxicity and oxidative stress-related lipid peroxidation in A549 cells in the ALI Vitrocell^®^ CLOUD system [22]. However, the exposure system used in this previous study could not be applied to distinguish the role of gas versus particle phase in DBP toxicity due to the restriction of using a solvent for liquid aerosol droplet generation. Therefore, the current study combines the knowledge on the toxic effects of the SVOC DBP on A549 cells at ALI with a newly designed system for the targeted analysis of gas versus particle toxicity.

## 2. Materials and Methods

### 2.1. Aerosol Generation

**Figure 1:**
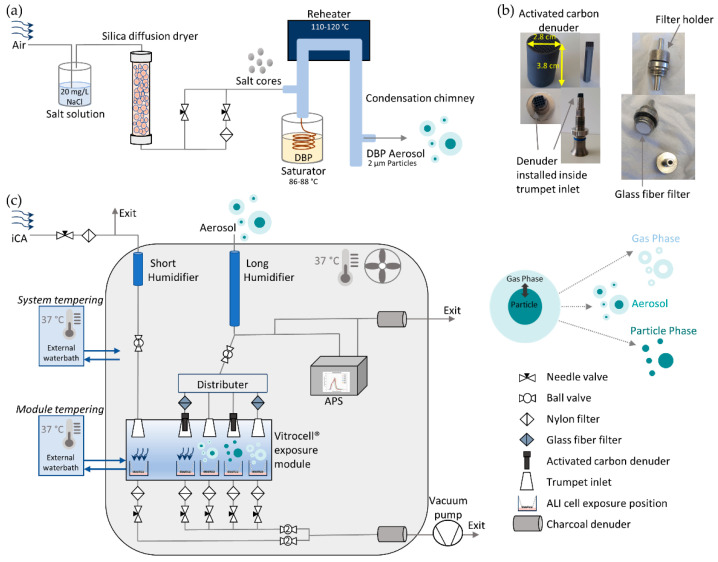
Detailed experimental set-up depicting the aerosol generation and in vitro ALI cell exposure system unit. (**a**) Scheme of the modified TOPAS SLG270 aerosol generator connected to the salt nucleation core production line. The salt cores are guided to the generator leading to formation of the liquid DBP (dibutyl phthalate) aerosol. (**b**) Activated carbon denuders are installed inside the trumpet inlet of the Vitrocell^®^ exposure module for pure particle phase exposures; glass fiber filters in manufactured holders are set upfront the trumpet inlet for pure gas phase exposures. (**c**) Detailed set-up of the exposure system including new exposure system and external components. APS = Aerodynamic Particle Sizer, iCA = independent clean air connection line.

A modified TOPAS SLG 270 aerosol generator (Topas GmbH, Dresden, Germany) was used for generating monodisperse particles with a mean aerodynamic diameter of 2 µm. A new salt (20 mg/L NaCl) nucleation core production line was set up in front of the generator body. The target compound DBP was flowed in the glass saturator jar of the generator. The temperature of the saturator as well as the flow rate through the saturator defines the amount of vapor that enters the system and therefore the amount that condenses on the salt nucleation cores. The particles pass through the re-heater and re-condense on the salt cores in the condensation chimney on the way out, which results in a more homogenous condensation on the salt nuclei. The amount of vapor along with the salt core concentration will define the size and concentration of the generated particles. The saturator temperature was set between 86 and 88 °C and the re-heater between 110 and 120 °C. A detailed scheme of the aerosol generation unit is seen in Figure 1a.

### 2.2. Exposure System Set-Up

A common fridge housing was modified to a climate chamber. The temperature in the climate chamber is regulated with an external waterbath, which guides warm water through a cupper heat exchanger inside the climate chamber, and a strong ventilator, which homogeneously distributes the heated air in order to maintain a constant temperature of 37 °C and prevent condensation. The new exposure system was then set up inside the climate chamber. An additional external waterbath set to 37 °C was installed for an independent temperature control of the Vitrocell^®^ exposure module (Vitrocell^®^ GmbH, Waldkirch, Germany) to guarantee optimal conditions for the cells. Clean, particle-free compressed air was generated with a scroll compressor (Atlas Copco Kompressoren und Drucklufttechnik GmbH, Essen, Germany) attached to a catalytic air purifier (Aadco instruments, Inc., Village of Cleves, OH, USA).

For separate exposures to gas and particle phases, glass fiber filters with high particle retention and wet strength (1.0 µm, 13 mm, binder free, Type A/E, SKC Inc., Eighty Four, PA, USA) and squared structured denuders consisting of activated carbon and ceramic (62 channels/cm^2^, Mann+Hummel Molecular filtration, Gefrees, Germany) were used, respectively. To minimize evaporation of particle phase after removal of the gaseous fraction, the denuders were installed as close as possible to the ALI exposure cell layer. Therefore, the dimensions of the denuders were adjusted to fit inside the Vitrocell^®^ module trumpet inlets (Figure 1b). Afterwards, any organic remainders on the denuders were removed in a gas chromatography oven under continuous nitrogen gas supply for 2 h at 200 °C. For pure gas phase exposures, special filter holders were crafted in our workshop. The holders harboring the glass fiber filters were installed directly in front of the Vitrocell^®^ module trumpet inlet (see Figure 1b,c).

The aerosol flow is guided to the new system, where it passes the humidification unit, is split into two separate tubing channels (one for exposure and one for particle online measurements), and subsequently equally distributed among four positions in the Vitrocell^®^ module. The first position receives a denuder (particle phase exposure), the second a filter (gas phase exposure), the third no installation (aerosol exposure), and the fourth with filter and denuder in series (clean air (CA) control). Additional independent clean air (iCA) receives an independent clean air supply line with an independent humidification unit. A detailed description of the exposure system is shown in Figure 1c. To achieve a relative humidity (RH) > 90% for cell exposures, two humidification units were installed inside the chamber. Both humidification units consisted of glass tubes with two lateral pipe attachments connected to a 250 mL Schott flask, which served as an ultrapure sterilized water reservoir (Milli-Q^®^, Merck, Germany). A centrifugal water pump (M400, RS PRO, RS Components, Frankfurt, Germany) continuously supplied water to the humidification units. A dialysis membrane (Spectra/Por^®^ 4 Dialysis Membrane, MWCO: 12–14 kD, diameter 6.4 mm, Standard RC Tubing, Spectrum LabsTM, San Francisco, CA, USA) was installed inside the glass tubes through which the airflow was guided and consequently humidified. One glass tube served for the humidification of the iCA (short humidifier glass tube, 20 cm length, ID 8 mm) and the other for the humidification of the aerosol flow (long humidifier glass tube, 65 cm length, ID 8 mm). The RH and temperature were directly measured at the Transwell^®^ insert positions inside the exposure module with sensors purchased from Vitrocell GmbH. Additionally, the denuders and filters for gas/particle discrimination of DBP were humidified at least 30 min prior to use in the exposure system.

Each cell position of the Vitrocell^®^ module received a perpendicular flow of 100 mL/min, which was guided with Iso-Versinic^®^ tubing. These tubes are generally used in Vitrocell^®^ ALI systems due to their conductivity, classification as USP-class VI, and biocompatibility according to ISO 10993. An aerodynamic particle sizer (auxiliary flow 1 L/min, APS, 3321, TSI Inc., Aachen, Germany) was installed inside the climate chamber after the humidification unit to measure particle size distribution and particle number concentration during exposures. For the aerosol-exposed positions, a four-way distributer (TSI Inc., Aachen, Germany) equally distributed the aerosol flow. The respective flow rates of 100 mL/min were adjusted with flow control needle valves. At each exhaust position of the Vitrocell^®^ module a nylon filter was installed upstream of the flow control valves. Two charcoal filters were installed at the aerosol exit lines to prevent contamination of the lab environment. The pressure gauge of the vacuum pump was set to −0.8 bar. To prevent possible leakage of the system, the tightness of the tubing system was verified with a negative pressure manometer. Two ball valves were installed that allowed for an easy handling of the cell exposures: the first one was set before the flow distributer (after the humidification unit), and the second one was installed after convergence of the individual exhausts of each cell exposure position before the vacuum pump. The iCA was controlled with two independent ball valves. For details of the climate chamber and the installed exposure system, see Figure 1c.

### 2.3. Activated Carbon Denuder Transmission

A modified Gormley–Kennedy equation for cylindrical denuders was used to estimate the theoretical working range of the activated carbon denuders [23,24,25] to guarantee minimal particle loss and nearly complete removal of gaseous DBP. The adsorption respective transmission efficiency is a function of the flow rate and denuder length. Size-dependent particle diffusion coefficients, as well as SVOC diffusion coefficient and vapor pressure are the component parameters controlling transmission/adsorption processes. The gas adsorption and particle transmission efficiency were previously validated with complete cylindrical denuder by using toluene as vapor phase component and hexadecane droplets with 2 µm hexadecane droplets as 2µm particles, respectively.

### 2.4. Exposure of Human A549 Cells at the Air–Liquid Interface (ALI)

Human A549 adenocarcinoma cells were purchased from the American Type Culture Collection (ATCC CCL-185). Cells were cultured in DMEM/F-12 + GlutaMAX^TM^ medium (Gibco, Paisley, UK) supplemented with 5% inactivated fetal bovine serum (FBS, Sigma-Aldrich, St. Louis, MO, USA), and 100 U/mL penicillin–100 µg/mL streptomycin (Sigma-Aldrich, St. Louis, MO, USA). A total of 300.000 cells were seeded on a porous polyester membrane of 6-well Transwell^®^ inserts (Product Nr. 3450, 24 mm diameter, 0.4 µm pore size, Corning, Kennebunk, ME, USA). Twenty-four hours after seeding, cells were further cultured at ALI for 24 h, and afterwards exposed to DBP (Sigma-Aldrich, St. Louis, MO, USA) over a 4 h period in the new exposure system. Briefly, cells grown on Transwell^®^ supports were placed into the Vitrocell^®^ exposure module inside the exposure system (37 °C) containing exposure medium consisting of the complete medium without FBS supplemented with 15 mM 4-(2-Hydroxyethyl)Piperazine-1-Ethanesulfonic Acid (HEPES, Gibco, Paisley, UK). A negative control kept in exposure medium in the incubator without CO_2_ during exposure was used in each experiment as a reference to distinguish possible toxic effects derived from the exposure procedure. After exposure, cells were directly harvested for toxicological analysis or post-incubated for further 20 h with complete medium at 37 °C and 5% CO_2_ at ALI until further analysis. Positive controls were used according to the performed assay.

### 2.5. Toxicity Evaluation

#### 2.5.1. Cytotoxicity, Cell Viability, and Live Cell Imaging Assays

Potential cytotoxic effects of the new exposure system itself—together with the applied installations of denuders and filters —as well as the SVOC compound DBP were evaluated by several assays. First, the new exposure system was evaluated with clean air exposures. Live cell imaging gave an overview on the integrity of the ALI cell layer and was conducted as a qualitative control of the Resazurin assay (see below). Cells were stained with 5 µg/mL bisbenzimide Hoechst 33342 trihydro-chloride (H 33342, Sigma-Aldrich, St. Louis, MO, USA) to stain the nuclei and 2.5 µg/mL propidium iodide (Biotium, Fremont, CA, USA) to selectively stain dead cells. Pictures were taken with a fluorescence microscope (BioTek, Lionheart FX, 4× magnification) with the respective fluorescence filters. For Hoechst staining, a DAPI filter (excitation/emission wavelength 377/447 nm) was used, and an RFP filter (excitation/emission wavelength 531/593 nm) was used for PI staining. After exposure, cytotoxicity and cell viability were evaluated in terms of lactate dehydrogenase (LDH) release and cellular metabolic activity with a resazurin solution. Lactate dehydrogenase (Cytotoxicity Detection Kit, Roche, Mannheim, Germany) and Resazurin (CellTiter-Blue^®^ Cell Vialbility Assay, Promega, Madison, WI, USA) assay were performed according to the manufacturer’s instructions. To determine the LDH release, the basolateral medium as well as the apical cell wash with Hanks’ Balanced Salt Solution (HBSS, Gibco, Paisley, UK) were collected. Cells treated with 2% Triton X-100 (Sigma-Aldrich, St. Louis, MO, USA) for 20 min before harvest served as positive controls and were used to determine the maximum LDH release. Normalization to the positive control gave the percentage of cytotoxicity. Resazurin assay was performed after exposures by incubating negative control and exposed cells with 10% resazurin solution in complete medium at 37 °C and 5% CO_2_ in a humidified incubator. Absorbance for LDH was detected with measurement/reference wavelengths of 493/620 nm and fluorescence for Resazurin assay was measured at 565/590 nm with a Thermo Scientific™ Varioskan™ LUX multimode microplate reader (Thermo Fisher Scientific, Schwerte, Germany).

#### 2.5.2. Evaluation of DNA Damage via Alkaline and Enzyme Version of Comet Assay

The mini-gel version of the alkaline comet assay was performed according to a previously published method [26] to detect DNA damage derived from strand break lesions. A detailed description of the procedure and buffers of the alkaline comet assay can be found elsewhere [22]. Additionally, the enzyme version of the comet assay with Formamidopyrimidine DNA glycosylase (Fpg) was conducted (4040-100-FM, Fpg FLARE™ Assay Kit, Trevigen, Gaithersburg, MD, USA) according to Di Bucchianico et al. (2017). Briefly, exposed A549 cells and controls were harvested by trypsinization (0.25% Trypsin–EDTA, Sigma-Aldrich, St. Louis, MO, USA) and diluted to a final concentration of 250.000 cells/mL. Two different positive controls were used: Cells treated with 30 μM hydrogen peroxide (H_2_O_2_) (EMSURE^®^ Merck, Darmstadt, Germany) for 5 min served as the positive control for the alkaline comet assay, while cells treated with 1.5 mM potassium bromide (KBrO_3_) (Acros Organics, Geel, Belgium) for 90 min served as the positive control for the enzyme version of the comet assay as suggested previously [27]. Mini-gels on microscopy slides were created with 1% low-melting-point agarose (Sigma-Aldrich, St. Louis, MO, USA). After 1 h of lysis, slides for the enzyme version of the comet assay were washed with washing buffer over 15 min. Afterwards, the samples were either subjected to Fpg enzyme (1:5 diluted) or FLARE buffer treatment for 30 min at 37 °C in a small chamber with a humid tissue. Slides for the alkaline comet version were kept in the lysis solution during this step. Finally, all slides were subjected to 40 min of alkaline unwinding and subsequent electrophoretic separation of 25 min (270–300 mA, 1.2 V/cm^2^). After neutralization, the slides were air-dried at least overnight. Comet pictures of the stained nucleoids (1:10,000 SYBR™ Gold Nucleic Acid Gel Stain (Invitrogen, Eugene, OA, USA)) were taken with a LionheartFX fluorescence microscope (10× magnification, BioTek, Germany). CometScore 2.0 software (TriTek Corp) was used to manually score at least 100 nucleoids per sample. Two replicate gels per sample were made (≥ 50 nucleoids per mini-gel scored) and at least three independent biological replicates were represented for statistical analysis. The results were depicted as mean % DNA in tail corresponding to the mean of the mean replicate mean ± SD.

### 2.6. Modelled and Measured DBP Deposition

**Figure A1:**
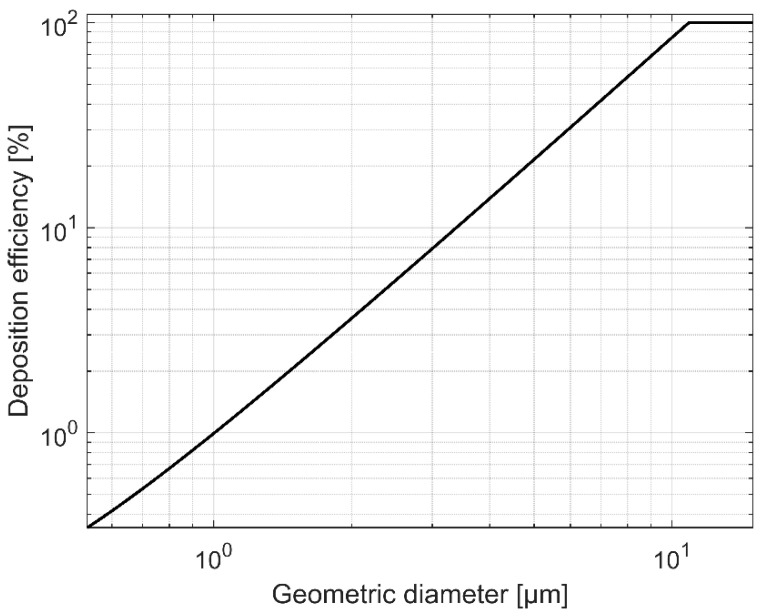
Calculated deposition efficiency [%] in the ALI exposure system as a function of particle geometric diameter (µm).

The deposited particle mass per area in Vitrocell^®^ ALI exposure systems is calculated with Equation (1),

deposited mass per area = (η × Q × N × T × *ρ* × V_p_)/A (1)

in which η is the deposition efficiency, Q is the aerosol flow, N is the particle number concentration (particle count per volume), T is the duration of the exposure, *ρ* is the particle density, A is the area of the deposition plate, and V_p_ is the particle volume assuming spherical particles. The size-dependent deposition efficiency of particles η in ALI exposure systems is calculated using the theory described in [17] and is a function of various parameters, including particle size and density, aerosol flow, geometry of the system, temperature, and pressure conditions in the system. The calculated deposition efficiency as a function of particle diameter is given in Appendix A Figure A1. In the model, the deposition of particles is controlled by diffusion and sedimentation mechanisms. Small particles deposit by diffusion and large particles by sedimentation. The particle number concentrations during exposures were measured by an APS. Density of DBP particles was set to 1.05 g/cm^3^. Density influences the calculations in two ways. Firstly, in determining the deposition efficiency and secondly and most critically in changing the deposited number of particles to deposited mass. The area of the deposition plate (ALI cell layer) was set to 4.7 cm^2^ according to the surface area of the 6-well Transwell^®^ inserts used (see Section 2.6). The aerosol flow and temperature over each position were set to 100 mL/min and 37 °C, respectively. This method was used in previous studies to calculate the deposited dose [28,29,30].

The experimental analysis of DBP deposition on the ALI cell layer was performed according to the following procedure. Immediately after cell exposure, the Transwell^®^ inserts were transferred to 6-well Nunclon^TM^ plates kept on an ice-cold metal cooling block. Quickly afterwards, ice-cold methanol (LC–MS-grade, Sigma-Aldrich, St. Louis, MO, USA) was added to the apical compartment of the inserts, followed by addition of the internal standard Phthalic acid 3,4,5,6-d4-dibutyl ester (DBP-d4, Sigma-Aldrich, St. Louis, MO, USA). Cells were scraped off and the whole cell suspension was collected, followed by a wash of the insert membrane with methanol, resulting in a final DBP-d4 concentration of 1 µg/mL. The samples were extracted in an ultrasonic bath for 3 min and centrifuged at 10,000× *g* for 5 min to get rid of remaining cell debris. The supernatant was directly transferred to HPLC vials (Agilent Technologies, Santa Clara, CA, USA) and stored at −80 °C until analysis via liquid chromatography tandem mass spectrometry (LC–MS/MS). The LC–MS/MS system comprises an Agilent 1290 UHPLC (Agilent Technologies, Santa Clara, CA, USA) including a degasser, a binary pump, an autosampler, and a column compartment, coupled to an Agilent 6470 MS/MS system equipped with an ESI Source. The amount of deposited DBP was measured for three independent experiments. A Kinetex C18 column (2.6 μm, 100 × 3 mm i.d., Phenomenex, Macclesfield, UK) was used for separation and the column compartment was set to 20 °C. A sample volume of 5 µL was injected at 20 °C. The measurement was performed in multiple reaction monitoring in positive ion mode using a gradient separation starting with 50% 0.1% acetic acid (mobile phase A) and 50% methanol (mobile phase B) with a constant flow of 300 µL/min going up to 100% methanol in 15 min. DBP m/z 279 → 149 and DBP-d4 m/z 283 → 153 transitions were used for quantification, and DBP m/z 279 → 205 and DBP-d4 m/z 283 → 209 transition ions served as qualifier ions. For quantification, an external calibration curve with DBP from 12 to 500 ng/mL was established.

### 2.7. Statistical Analysis

Statistical analysis was performed with GraphPad Prism 5. Statistical significances in the results were calculated by one-way analysis of variance, followed by Tukey’s multiple comparison post-hoc test. All comparisons were considered significantly different when *p* was <0.05. Data are shown as mean ± SD.

## 3. Results

### 3.1. Aerosol Generation

**Figure A2:**
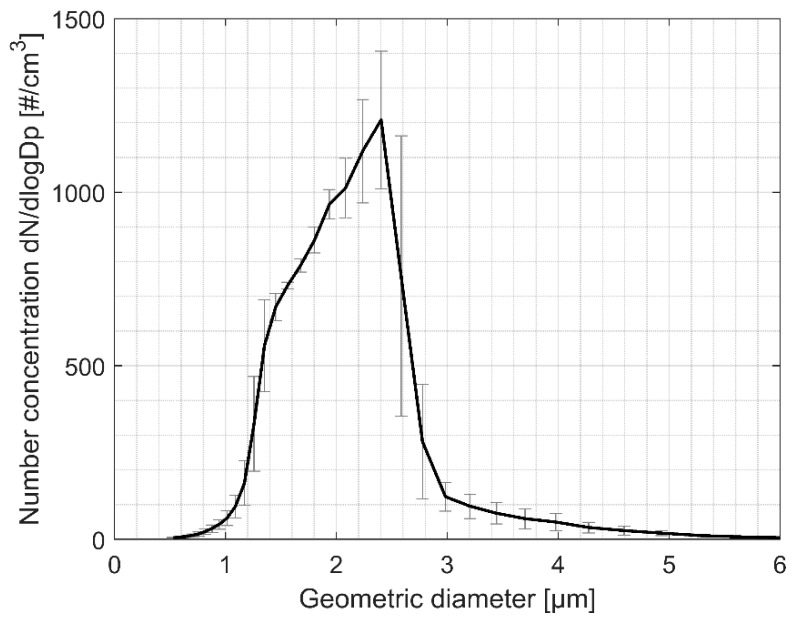
Dibutyl phthalate particle number concentration dN/dlogDp [#/cm^3^] ± SD generated with a mean particle geometric diameter of 2.0 ± 0.05 µm SD with the modified TOPAS SLG270 aerosol generator. The aerodynamic diameter measured by APS is converted to the geometric diameter using the density of DBP.

The particle size distributions were measured online by APS during each exposure. A geometric mean diameter of 2.0 ± 0.05 µm with a mean number concentration of 320 ± 8 #/cm^3^ was measured (see Appendix A Figure A2). The gas phase mass concentration was calculated as a function of temperature using the ideal gas law. The gas phase concentration depends strongly on the vapor pressure value. Unfortunately, fit curves for vapor pressure data obtained at higher temperatures are not always as accurate as for lower temperatures. No experimental data are available for the vapor pressure of DBP at 37 °C. In previous studies, a curve was fitted to available experimental data for DBP at different temperatures. The curve reflects the relationship between the temperature and the vapor pressure of DBP based on experimental data [31,32]. The extrapolated value of the DBP vapor pressure at 37 °C is about 4.5 times increased compared to its vapor pressure value at 25 °C. The goal was to achieve a nearly balanced mass concentration of the gas and particle phases. With available extrapolated vapor pressure values, the gas phase mass concentration of DBP at 37 °C amounts to about 1.8 mg/m^3^, which corresponds to approximately 50% of the total mass. The gas phase mass concentration amounts to about 0.42 mg/m^3^ at 25 °C, corresponding to about 18% of the total mass. On the other hand, the empirical measurements of the particle–vapor distribution for DBP at 35 °C and 1.2 mg/m^3^ were close to 50% [33]. Due to the lack of precise information about DBP vapor pressure at 37 °C, we can only estimate that the particle and gas phase concentrations were equal during the exposures. The mean particle mass concentration during the exposures resulted in 1.86 ± 0.06 mg/m^3^.

### 3.2. Performance Evaluation of the New System

**Figure A3:**
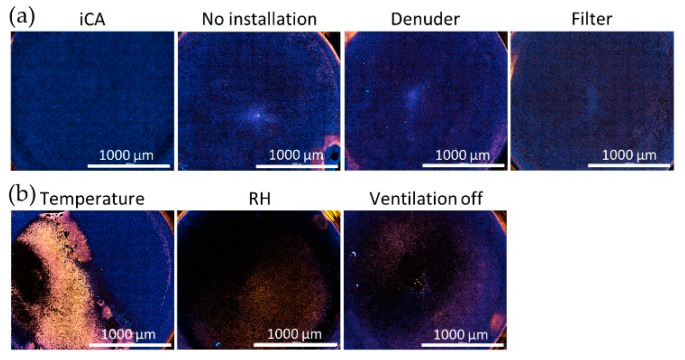
Optimization of parameters crucial for the integrity of the ALI monolayer during cell exposures. (**a**) Intact ALI cell layer when all parameters are accordingly adjusted. Neither activated carbon denuder, nor glass fiber filter installations affect the ALI cell layer. (**b**) From left to right: ALI cell layer impairment if no external module tempering is applied, RH % is insufficient, and the ventilation engine is turned off.

The newly designed exposure system illustrated in Figure 1 allowed for the safe operation and handling of cell exposures due to the tight assembly of the single units and the simple tubing alignment. The system was evaluated with clean air during 4 h and 24 h exposures. Appendix B (Figure A3a) shows that the integrity of the ALI cell layer is maintained during 4 h exposures under the applied conditions. The critical factors for cell exposures in the newly designed system identified during the conducted experiments included the following:

1. Temperature: The heating and ventilation of the climate chamber as well as the independent temperature control of the Vitrocell^®^ exposure module ensured optimal temperature conditions for cell exposures (37 °C) that prevent condensation. The temperature within the climate chamber varied between ±0.2 °C, while the variation in the exposure module was about ±0.1 °C. Appendix B Figure A3b reveals the importance of a constant temperature of the exposure module and a homogeneous heat distribution in the modified climate chamber. Indeed, the cell monolayer is severely disrupted without external module heating, leading to cell detachment on half of the membrane support. If no ventilation homogeneously distributes the heat, condensation may occur that leads to droplet deposition on the ALI cell layer, causing cell detachment in the center of the membrane.
2. Relative humidity: The humidification units (short and long humidifier) provided a RH > 90% for iCA and the aerosol-exposed positions, allowing for the sufficient humidification of the cells exposed at ALI as measured within each single exposure module position. The humidified activated carbon and ceramic denuders and the glass fiber filters—allowing for particle versus gas phase exposure—did not impair the humidification in the exposure module. Without the adjustment of the RH, the incoming airflow dries the cells out, leading to cell death and detachment (Appendix B Figure A3b).
3. Airflow: The flow rate at each position was adjusted with flow control needle valves at 37 °C to 100 ± 10 mL/min.
4. Air–liquid interface cell layer integrity and cell survival: Purified dry air was applied to the independent clean air (iCA) as well as the four exposure positions with filter and/or denuder installed for checking any potential impairment of the ALI cell monolayer induced by the cell exposure system. In comparison to an external incubator control, no significant effect was observed caused by the incubation system. The detaching of cells (compare Appendix B Figure A3a) was not discernible for the 4 h experiments.

**Figure A4:**
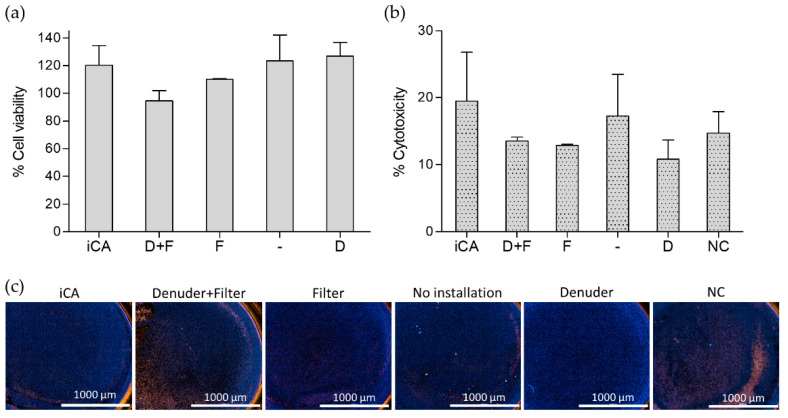
Effect of the new ALI exposure system with its different installations on A549 cells upon 24 h exposure to clean air (*n* = 2). (**a**) Percentage cell viability normalized to the NC, and (**b**) percentage cytotoxicity compared to the NC measured by normalization to the total LDH release (2% Triton X-100 positive control). (**c**) Live cell imaging with 5 µg/mL H 33342 to stain the nuclei (blue) and 2.5 µg/mL propidium iodide (red) to selectively stain dead cells. iCA = independent clean air, NC = negative control. Installations: F = Filter; D + F = Denuder + Filter; D = Denuder; No installation= (−).

Since 4 h exposures were successfully conducted, A549 cells were exposed for 24 h to clean air in order to demonstrate the possibility to perform long-term exposures and therefore extend the application spectrum of the new system. Appendix B Figure A4 depicts the effect of the system and its installations on the ALI cell layer after 24 h exposure. A slight decrease in cell viability appeared upon exposure to air with serial installed filter and denuder (Appendix B Figure A4a). The serial filter and denuder position was an exception, since all other clean-air-exposed cells showed a higher metabolic activity compared to the negative control (NC). This might be due to a higher number of dead cells observed in the NC with live/dead staining (Appendix B Figure A4c). However, cytotoxicity levels were comparable between the CA-exposed cells and NC (Appendix B Figure A4b). For clean-air-exposed positions with either filter or denuder, the endpoints to assess the integrity of the ALI cell layer revealed neither an increase in cytotoxicity, nor a decrease in cell viability.

### 3.3. Activated Charcoal Denuder Efficiency

The calculated removal efficiency of the activated carbon and ceramic denuders for gas phase DBP is more than 99%. This indicates that almost no gas phase of DBP is passing through the charcoal denuder at the chosen flow rate of 100 mL/min. The particle transmission efficiency of the new denuder was tested for small particles with soot and for larger particles with Hexadecane. Due to diffusion, the particle losses occurred for very small soot particles (<100 nm) at low flow rates. Large particles may impact on the denuder wall at high flows. For the DBP particles of about 2 µm, a transmission efficiency higher than 95% was measured with the applied denuders at 37 °C.

### 3.4. Toxicicity of DBP Gas versus Particle Phase versus Aerosol in A549 Cells

#### 3.4.1. Cytotoxicity, Cell Viability, and Live Cell Imaging

**Figure 2:**
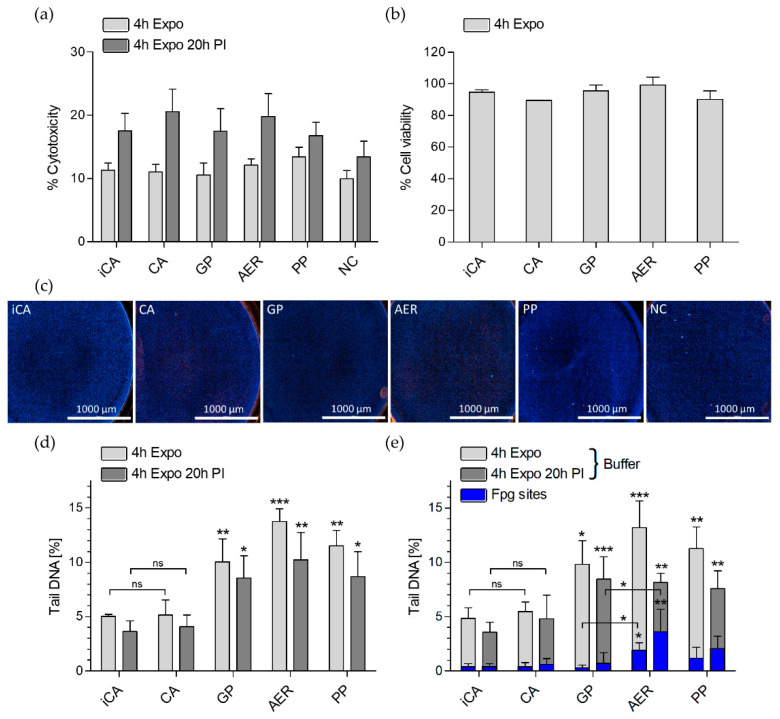
Toxicity assessment of DBP gas versus particle phase and total aerosol on human lung A549 cells exposed at ALI. A549 cells were exposed to DBP over 4 h with or without 20 h post incubation (4 h Expo or 4 h Expo 20 h PI). (**a**) % Cytotoxicity compared to the NC measured by LDH assay and normalized to the total LDH release (2% Triton X-100 positive control) (*n* ≥ 6), (**b**) % Cell viability measured by resazurin assay and normalized to the NC (*n* = 3), and (**c**) Live/dead cell imaging with 5 µg/mL H 33342 to stain the nuclei (blue) and 2.5 µg/mL propidium iodide (red) to selectively stain dead cells after 4h Expo to iCA, CA, or the different SVOC phases of DBP. (**d**) Genotoxicity assessed by comet assay with the alkaline version and (**e**) the enzyme version with Fpg and the respective Buffer controls (without Fpg) after the respective time points (*n* ≥ 3). iCA = independent clean air, CA = clean air, GP = gas phase, AER = aerosol, PP = particle phase, NC = negative control. Data are shown as mean  ±  SD. Significances are shown in comparison to the iCA of the respective time point (Statistical analysis via Tukey One-way ANOVA, * *p*  ≤ 0 .05, ** *p*  ≤  0.01, *** *p*  ≤  0.001).

Cell exposures in the new exposure system to (i-)CA, and the different SVOC phases of DBP showed no induction of cytotoxicity compared to the NC at the two time points—4 h after exposure with and without 20 h post-incubation (PI) (4h Exposure and 4h Exposure + 20h PI) (Figure 2a). In addition, the cell viability of the A549 cells was not affected 4 h after exposure (Figure 2b). Figure 2c depicts representative images of live/dead cell imaging 4 h after exposure to (i-)CA, DBP gas phase (GP), aerosol (AER), particle phase (PP), and the NC. The pictures show that the ALI cell layer maintains its integrity during exposures without cell detachment. The data indicate the suitability of the new system for conducting ALI cell exposures for assessing GP and PP toxicity of SVOCs.

#### 3.4.2. Genotoxicity

**Figure A5:**
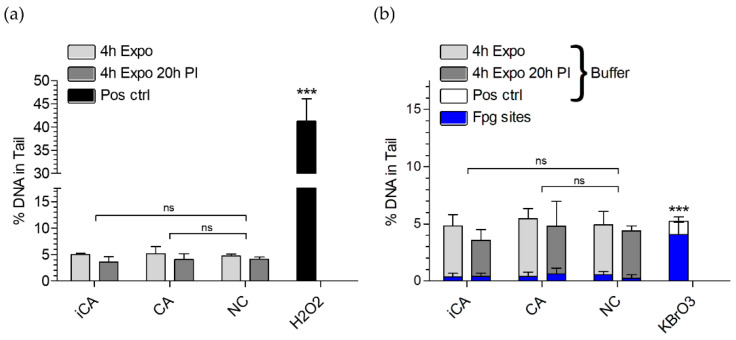
(**a**) Genotoxicity of negative ((i-)CA, NC) and positive controls (Pos ctrl) assessed by comet assay with the alkaline version (Pos ctrl 30 µM H_2_O_2_) and (**b**) the enzyme version with Fpg (Pos ctrl 1.5 mM KBrO_3_) and the respective Buffer controls (without Fpg) after the respective time points (*n* ≥ 3). iCA = independent clean air, CA = clean air, Data are shown as mean ± SD. Significances are shown in comparison to the negative controls (Statistical analysis via Tukey One-way ANOVA, *** *p*  ≤  0.001).

Genotoxicity was assessed under non-cytotoxic conditions at the respective time points (see Section 3.4.1) to avoid the confounding effects of DNA damage, which are potentially induced by increased cell death. Figure 2d,e shows the inferred DNA damage upon (i-)CA, DBP GP, AER, and PP exposure in A549 cells 4 h after exposure with and without 20 h PI. The basal DNA damage of the CA did not differ significantly from the iCA at both time points. The alkaline version of the comet assay (Figure 2d) and the buffer-treated samples of the enzyme version of the comet assay (Figure 2e) clearly indicate a significant increase in DNA strand break lesions upon treatment with the distinct physical states of DBP in comparison to the (i-)CA. No significant difference in the increased % DNA in tail could be observed between the different DBP exposure conditions at the respective time points. The Fpg enzyme treatment revealed significantly increased oxidative DNA damage upon AER treatment compared to (i-)CA and GP, but not PP (Figure 2e). More pronounced effects in terms of DNA strand break lesions and alkali labile sites were detected after 4 h exposure in comparison to cells exposed for 4 h followed by 20 h PI. In contrast, the potency of DBP to induce oxidative DNA damage in A549 cells was comparably higher after 20 h of post-incubation. The basal levels of the DNA damage of the (i-)CA were comparable to the NC in both comet versions (see Appendix B Figure A5). The alkaline positive control (30 µM H_2_O_2_) led to a significant increase in DNA damage (Appendix B Figure A5a), and the positive control of the enzyme version (1.5 mM KBrO_3_) selectively induced a significant increase in oxidative DNA lesions (Appendix B Figure A5b).

### 3.5. Calculated and Measured Deposition of DBP Particles

The deposited dose of particles in the ALI exposure module was calculated using the theoretical model described before and estimated to be about 680 ± 100 ng/cm^2^ over a 4 h exposure. The particle size distributions measured by APS used for these calculations are presented in Appendix A Figure A2.

**Figure 3:**
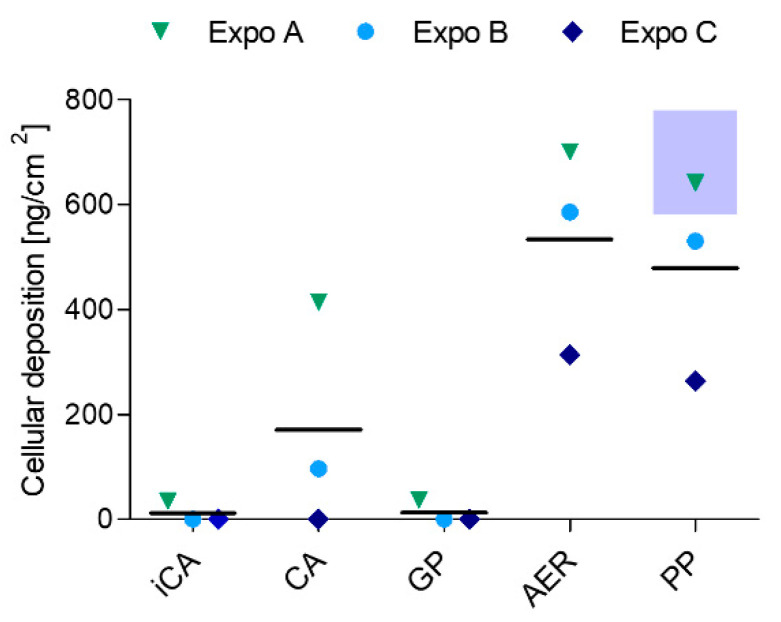
Liquid chromatography tandem mass spectrometry measurements of DBP deposition [ng/cm^2^] on the ALI cell layer upon exposure to the different SVOC phases of DBP in three independent exposures (Expos A, B, C). The blue shaded area represents the deposition range of the model calculations of DBP particles. iCA = independent clean air, CA = clean air, GP = gas phase, AER = aerosol, PP = particle phase.

Figure 3 shows that aerosol AER and PP encounter similar doses of deposited DBP on the ALI cell layer, with 530 ± 200 ng/cm^2^ AER and 480 ± 190 ng/cm^2^ PP, as measured by LC-MS/MS. The measured deposition was remarkably close to the calculated deposition of the DBP particles.

However, the GP deposition could not be measured and therefore was comparable to the iCA. While a high deposition in Expos A and B was observed, the deposition in Expo C was comparably low. The high deposition in the CA position (bearing filter and denuder installation in series) in Expos A and B (mean deposited dose 170 ± 217 ng/cm^2^) can either be explained by defective glass fiber filters or the inadequate situating of the filter inside the filter holder. Since no DBP can be observed in the only filter-installed position, and Expos A and B were conducted on the same day with different cell batches, this explanation is very reasonable.

## 4. Discussion

Our current study aimed to develop an exposure system for evaluating the toxicity of the different aggregation states of semi-volatile organic compounds (SVOCs) using dibutyl phthalate (DBP) as a model compound. The newly designed exposure system had to fulfill certain requirements including installations not affecting humidity or the integrity of the ALI cell layer, stable and homogeneous temperature conditions to avoid condensation, and a stable airflow. The importance of a preconditioned aerosol air flow with an adequate temperature and RH for ALI cell exposures was shown previously [34]. From a toxicological point of view, neither the activated carbon denuder, nor the glass fiber filter installation affected the ALI cell layer. Additionally, the CA position, which incorporated both installations, showed no genotoxic impact compared to the gas phase, aerosol, and particle phase-exposed positions and exhibited a similar basal DNA damage as the iCA, indicating that both installations efficiently remove the gas and particle phase fractions during exposure and guarantee exposure to clean air.

While the genotoxic effect of the distinct SVOC phases of DBP regarding induced single and double DNA strand breaks and alkali labile sites were comparable to each other, major differences between the different DBP fractions could be observed in terms of oxidative DNA damage. Aerosol exposure led to the highest increase in oxidative DNA damage and only the particle phase was inducing oxidative DNA lesions at a comparable level. Critically, the gas phase did not increase oxidative DNA damage compared to the (i-)CA, indicating that the genotoxicity induced by DBP in alveolar cells may mainly be attributed to the particle phase fraction. The oxidative DNA damage was found significantly increased in the aerosol after both time points and encountered an even higher increase after an additional 20 h of post-exposure, indicating a persistent oxidative stress induction by DBP. Though not significant, the same effect applied to particle phase exposure. It may hence be suggested that the gas and particle phases of DBP or any given SVOC may infer genotoxicity by distinct modes of action. This outcome in turn indicates that the physical state of SVOCs implies an additional crucial parameter in the prediction of hazard and exposure risk assessment, together with particle size-associated SVOC adsorption and total mass. The importance of considering gas phase SVOCs for risk assessment has been nourished by the high contribution of gas phase mass concentrations to total polycyclic hydrocarbon yield in the atmosphere [21].

It has to be noted that the generated particles represent droplets in a rather big size range characteristic for workplaces [35]. Given the fact that the toxic effects of a compound may rather be exerted by UFPs than fine particles or gas phase [15], the overall picture of the observed particle-induced toxic effects may change profoundly upon shifting the exposure to smaller particle size fractions, which would ultimately result in a higher particle number concentration to accomplish an equal mass concentration. To be able to compare the respective concentrations of the gas and particle phases, the mass concentrations of both phases were approximated to be equal during the conducted experiments. Further studies should hence incorporate an aerosol generation unit that allows for UFP exposures and a subsequent elevated local dose due to the introduction of solid particles rather than liquid droplets, which may generate a liquid film on top of the ALI cell layer. In this context, it would be helpful to switch to a distinct condensation core for the SVOC compound, such as soot [28]. In addition, the use of solid particles would allow for the visualization of the time-resolved transmission electron microscopy deposition and cellular uptake analysis.

With a geometric mean particle diameter of 2.0 ± 0.05 µm and a mean number concentration of 320 ± 8 #/cm^3^, the calculated particle deposition resulted in 680 ± 100 ng/cm^2^ over a period of 4 h exposure time. These model results are in excellent agreement with the LC-MS/MS measurements (530 ± 200 ng/cm^2^ for total aerosol and 480 ± 190 ng/cm^2^ for particles). However, the model cannot be used to calculate gas phase deposition. Particle phase exposure resulted in a minor decrease (~11%) in deposited DBP compared to the aerosol-exposed group. This reduction might correspond to the amount of gas phase. Since we implemented a relatively high stagnation flow rate of 100 ± 10 mL/min, the efficient delivery of the gas components with less variability can be assumed [18]. On the other hand, we were not able to quantify the deposited gas phase via LC-MS/MS, indicating that the deposited gas phase or its contribution to the total aerosol is rather small. According to the Henry’s law constant and the air/water partition coefficient of DBP reported in [36], we expect the gas phase of DBP to remain in the air rather than partitioning into the aqueous cell layer. In our initial estimation, the gas and particle phase mass concentrations were assumed to be equal. Indeed, we might have overestimated the gas phase mass concentration, since a saturation gas phase concentration of around 0.5 mg/m^3^ was previously measured at 35 °C [33], whereas in our calculations the saturation gas phase concentration amounted to around 1.8 mg/m^3^ at 37 °C. Besides, the gas phase may adsorb to the conductive tubing, resulting in a rather small gas phase fraction compared to the assumed calculated value. Additionally, gas phase exposure might result in an enhanced fraction of gas phase DBP reaching the cytosol, where the enzymatic conversion of esterases and unspecific lipases may readily result in the conversion of DBP to its monoester form monobutyl phthalate (MBP) [37]. Given the fact that the potency of MBP to induce DNA damage is decreased compared to the parental compound DBP [38], this might explain the absence of oxidative DNA damage induced by the gas phase. However, the small fraction of pure gas phase was still sufficient to inflict considerable DNA damage. This is coherent with our previous study, where even small amounts of DBP (0.02 to 20 ng/cm^2^) induced a significant increase in DNA strand lesions upon DBP exposure in A549 cells in the ALI CLOUD Vitrocell^®^ system [22].

While the modular set-up of the system follows the idea of current ALI systems [16], the simplicity of the newly developed system provides for several advantages. These include a straightforward aerosol flow preconditioning at homogeneous temperatures without the requirement of turbulent premixing in a heated reactor for humidification [39], which leads to reduced system-derived changes in aerosol physicochemical properties [16], the easy cleaning and maintenance of the new system, respective reuse with other emission sources by quick and simple tube exchange, as well as the easy implementation of the system in individual labs. It has to be mentioned that the first version of the new ALI system implements only one Vitrocell^®^ exposure module, corresponding to one technical replicate per exposure. To confirm repeatability and reproducibility, additional exposure modules should be installed, which in turn requires the adequate adjustment of the tubing alignment and aerosol distribution unit to guarantee equal exposure conditions in the respective module positions.

Our results indicate that the new exposure system can be used to investigate the distinguishing effects of the gas and particle phase toxicity of the compounds with higher volatility. For those compounds, gas phase exposure becomes more relevant in terms of inhalation toxicology. Hence, the gas phase concentration and vapor pressure of a given organic compound should be pre-experimentally validated to enable exposures to similar mass concentrations of gas and particle phase. For model evaluations, DBP reveals not be the optimal SVOC of choice due to its low volatility and resulting low gas phase contribution in the experiments. Nevertheless, the modular construction of the system allows for the selection of the distinct types of aerosol emission sources that represent more complex airborne material.

Additionally, 24 h exposures to clean air showed that it is feasible to perform exposures that consider the real exposure scenario, including the adequate time resolution. In combination with more advanced cell models, this application may significantly contribute to elucidating the pathophysiological mechanisms that initiate or promote disease-related processes occurring due to chronic exposure.

In addition, the online measurements of aerosol characteristics can be efficiently employed to estimate the deposited dose over longer exposure durations (4 h exposures), which is supported by the good agreement of the model calculations and the empirically measured particle deposition. Furthermore, the relatively high flow rates of 100 mL/min may result in an efficient deposition of gas phase pollutants on the ALI cell surface [18].

Most of the studies that investigate airborne toxicity by employing ALI exposure systems focus on particle-induced effects and respective deposition efficiency [16]. Recently, ALI systems were developed that address the toxicity and deposition efficiency of gaseous pollutants, including volatile organic compounds (VOCs) or trace gases (i.e., ozone, carbon monoxide, nitric oxide, etc.) [18,19,20]. Our newly developed system complements the available systems by the possibility to study the effects of both the gas and particle phases, as well as the total aerosol of airborne pollutants including VOCs and SVOCs. The consideration of both the gas and particle phases of chemical pollutants for toxicological risk assessment is critically important. Otherwise, airborne exposure and the associated potential adverse health effects may be severely underestimated [14,21]. In the future, the current ALI system can be employed to attribute potential adverse health effects to the specific aerosol composition of gaseous- and particle-associated airborne pollutants and consider the toxicological effects that derive from both physical states.

## 5. Conclusions

To evaluate the potential distinguishing effects of the gas and particle phases of SVOCs, a new in vitro ALI exposure system was developed. The new approach allowed for separate exposures to the respective SVOC aerosol phases and the concomitant evaluation of the deposited particles—with congruent deposition results of the calculated estimates and empirical data. Genotoxic investigation of the compound DBP revealed that induced oxidative DNA strand break lesions in human A549 cells exposed at ALI may be a result of particle-induced effects rather than the gas phase, and hint at the distinct features of toxicity induction based on the aggregation state of SVOCs. Since cells are surviving and not detaching over an exposure period of 24 h, it can be concluded that the system is generally suitable for long-term exposures. Within this scope, prospective studies with long-term exposures and the use of cell models more representative of the human airways may further help to adapt the concept of in vitro ALI exposures to represent more realistic exposure conditions.

## Data Availability

Not applicable.
